# Gastrointestinal stromal tumour of the rectum: a report of two cases

**Published:** 2012-06-01

**Authors:** Tarik Chekrine, Hassan Jouhadi, Zineb Bouchbika, Nadia Benchakroun, Nezha Tawfiq, Souha Sahraoui, Abdelatif Benider

**Affiliations:** 1 Service d'Oncologie-Radiothérapie, Centre Hospitalier Universitaire Ibn Rochd, 1 quartier des hôpitaux, 20360 Casablanca, Maroc

**Keywords:** Rectum, gastrointestinal stromal tumour, diagnosis, treatment

## Abstract

Gastrointestinal stromal tumours (GISTs) are the most common mesenchymal tumours of the gastrointestinal tract in adults, although rectal localisation of these tumours is very rare. We report here two cases of rectal stromal tumours in a 77-year-old woman and a 65-year-old man, confirmed by histology and immunohistochemistry. Surgery for rectal GIST patients is the standard treatment and adjuvant imatinib, a tyrosine kinase inhibitor, is indicated for GISTs with a high risk of malignancy, as well as in the case of metastatic or unresectable tumours.

## Introduction

Gastrointestinal stromal tumours (GISTs) are the most frequent mesenchymal gastrointestinal tract tumours and are characterised by an overexpression of a tyrosine kinase (c-Kit or CD117) [1]. They usually develop in the stomach lining (70%), the small intestine (20 to 30%), and, in rare cases, the colon, rectum or oesophagus. GISTs with rectal localisation account for only 0.1% of all rectal tumours [2]. We report here two cases of rectal stromal tumours, one of them presenting with liver metastases.

## Patients and case report

### Case 1

**Figure 1:**
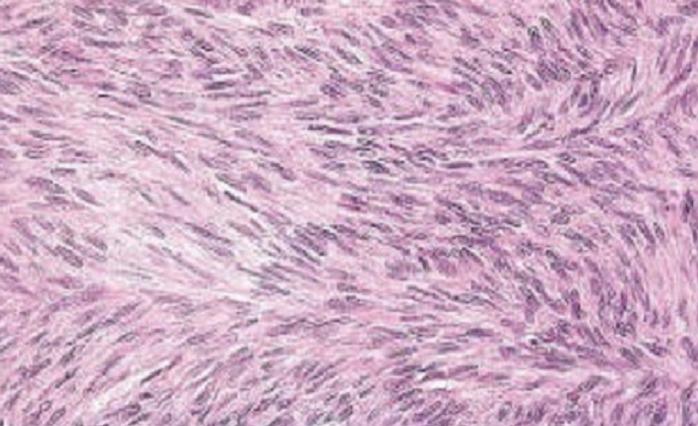
Microscopic appearance of the rectal stromal tumour composed of spindle cells

A 65-year-old man presented to the hospital in March 2006 with a rectal syndrome with minimal rectal bleeding, which had appeared two months earlier, and with an overall deterioration of health. The patient had a history of cholecystectomy. A complete proctologic examination revealed a left lateral mass 4 cm from the anal margin, with a good sphincter tone. A biopsy was performed. The anatomopathological study showed morphological and immunohistochemical aspects, suggesting a spindle cell GIST (Figure 1). X-ray computed tomography (CT) of the abdomen and pelvis revealed the presence of a rectal tumour mass of 6 cm in diameter, pre-sacral and well encapsulated. Proctectomy and coloanal anastomosis were performed. After a histopathologic examination of the resected specimen, the diagnosis of malignant stromal tumour was made (intense expression of the anti-c-kit antibody; size > 5 cm; high mitotic index; mitosis > 5/50 fields). The postoperative course was uneventful. Eight months after surgery, the patient presented with multifocal, unresectable liver metastases, for which he was treated with imatinib. The hepatic lesions remained stable 12 months after treatment onset. Treatment was then suspended due to the patient's general health deterioration and tumour progression.

### Case 2

**Figure 2:**
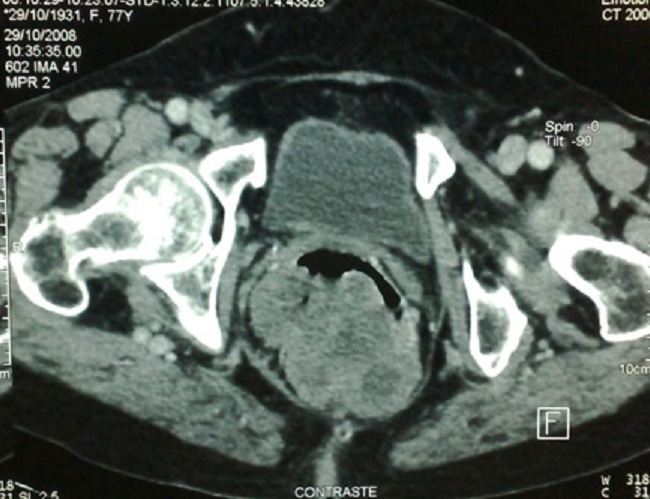
Pelvic CT scan showing the rectal lesion

A 77-year-old woman without significant medical history presented to the hospital in October 2008 with rectal tenesmus and bleeding of medium abundance, which had appeared two months earlier. Digital rectal examination revealed a deep indurate sessile mass located 3 cm from the anal margin and leading up into the lower rectum. A proctoscopy revealed a tumour process extending from the dentate line and 10 cm from the anal margin. A biopsy was performed. The histopathologic study showed morphological and immunohistochemical aspects, suggesting a GIST. A colonoscopy did not reveal abnormalities in other parts of the colon. Thoracoabdominal and pelvic CT scans were then performed for staging. This examination provided an objective view of the lesion, showing a rectal tissue tumour of 8 cm in diameter, located on the posterior wall and showing an infiltration of presacral fat, but without bone involvement (Figure 2). No lymph node, liver or lung injury was detected. An abdominoperineal resection was performed. The histopathologic study revealed a submucosal, ulcerated tumour, measuring 8 x 5 x 5 cm, and consisting of a rather dense fuso-cellular proliferation with fascicular architecture, a high risk of malignancy (mitosis > 5/50 fields, tumour size > 5 cm), and a complete resection. The immunohistochemical study showed a diffuse and intense c-kit and CD34 expression, confirming the stromal tumour type, whereas the other differentiation markers were negative (desmin and actin). The postoperative course was uneventful. The patient received adjuvant treatment with imatinib for a year, given the potential malignant aspect of the tumour. The evolution was good 13 months later.

## Discussion

Stromal tumours have been defined since 1998, with the discovery of the c-kit proto-oncogene mutation, and can develop along the entire gastrointestinal tract, with a decreasing frequency from the stomach to the rectum. Rectal localisation is rare, and rectal stromal tumours are more frequent in men, with a mean age of 60. The male and female patients in this report were aged 65 and 77, respectively.

The symptoms and diagnoses of rectal GISTs do not differ from other rectal tumours by digital rectal examination, proctoscopy, colonoscopy, and rectal endoscopic ultrasonography (EUS) with biopsy [3]. EUS is the ‘gold standard’ examination by which to confirm the parietal nature of the tumour, and to specify the layer from which it develops [4].

GISTs typically express c-kit, CD34, the smooth muscle antigen (SMA) and S100, but the expression of these factors vary according to the localisation of the tumour [1, 3, 5]. Our patients showed an intense overexpression of c-kit. Miettinen et al. showed that CD34 was expressed in 92% of rectal GISTs against only 50% of small intestine GISTs [5]. Alternatively, SMA was expressed in 1 to 4% of rectal GISTs vs. 47% of small intestine GISTs. The reason for these variations has not yet been elucidated.

An abdominopelvic CT scan or an MRI can assess the locoregional extension of the tumour and detect the presence of any metastasis, particularly in the liver [6].

The risk of malignancy depends on the tumour's size and mitotic count. These two criteria are also the base of the Fletcher prognostic scale (ranging from very low, to low, to intermediate, and to high risk) [1]. A mitotic index over 5/50 fields with high growth, or a tumour size over 5 cm are considered unfavourable prognostic factors [1]. The anatomical localisation has been proposed as a prognostic factor independent from the tumour size or mitotic index. In this regard, small intestine GISTs show a poorer prognosis, whereas other authors considered oesophageal and colorectal lesions as malignant [5].

The preferred treatment for GISTs remains surgery, as it is the only potentially curative treatment when performing a complete R0 resection without invasive tumour, and with clear margins [6, 7]. Lymph node dissection is not necessary, because lymph node metastases are rare (<10% of cases) [7]. Several surgical procedures are possible: local endoscopic excision with safety margin, rectal anterior resection, and abdomino-perineal removal, depending on the size and localisation of the tumour [4].

C-kit is the target of the molecule "imatinib mesylate,” a selective inhibitor of the tyrosine kinase receptor. Its neoadjuvant use can improve resectability or avoid mutilating surgery of aggressive and locally advanced GISTs [8]. Imatinib is also used as adjuvant after surgery for high-risk tumours or in cases of incomplete resection [3]. This molecule remains the only treatment for aggressive, locally advanced, inoperable and/or metastatic tumours [3]. Conventional chemotherapy and radiotherapy have not proved effective and are no longer used [7]. Post-treatment surveillance of localised stages is based on clinical examination, abdominal and pelvic CT scans, and rectal EUS. Relapses mainly occur under the diaphragm or in the liver and/or the peritoneum. Overall, the literature reports five-year survival ranges from 28 to 60% [6].

## Conclusion

In conclusion, rectal localisation of GISTs is rare. For localized tumours, surgical resection is the preferable treatment. Imatinib, a tyrosine kinase inhibitor, is indicated as an adjuvant for GISTs with a high risk of malignancy, as well as for metastatic or unresectable tumours.
